# Relationships between the Mini-InDel Variants within the Goat *CFAP43* Gene and Body Traits

**DOI:** 10.3390/ani12243447

**Published:** 2022-12-07

**Authors:** Fang Mi, Xianfeng Wu, Zhen Wang, Ruolan Wang, Xianyong Lan

**Affiliations:** 1 Institute of Animal Husbandry and Veterinary Medicine, Fujian Academy of Agricultural Sciences, Fuzhou 350000, China; 2 College of Animal Science and Technology, Northwest Agriculture and Forestry University, No. 22, Xinong Road, Xianyang 712100, China

**Keywords:** goat, *CFAP43*, body traits, InDel (insertion/deletion), correlation analysis

## Abstract

**Simple Summary:**

The cilia- and flagella-associated protein 43 (*CFAP43*) gene plays an important role in the formation of flagella and cilia, and limited research has been conducted on the effect of this gene on animal body traits. Herein, the relationships between three insertion/deletion polymorphisms (L-13, L-16, and L-19) of the goat *CFAP43* gene and body traits were analyzed in 1827 Shaanbei white cashmere goats. The findings showed that L-13 was related to chest depth; L-16 was associated with body length, chest width, cannon circumference, and back height; L-19 was associated with body length, chest circumference, and cannon circumference. The ideal genotypes for most of these traits are the deletion/deletion or insertion/deletion genotypes, which is consistent with previously published ideal genotypes for litter size at these loci; therefore, these loci are expected to be molecular markers for improving both body traits and litter size traits.

**Abstract:**

The cilia- and flagella-associated protein 43 (*CFAP43*) gene encodes a member of the cilia- and flagellum-associated protein family. Cilia on the cell surface influence intercellular signaling and are involved in biological processes such as osteogenesis and energy metabolism in animals. Previous studies have shown that insertion/deletion (InDel) variants in the *CFAP43* gene affect litter size in Shaanbei white cashmere (SBWC) goats, and that litter size and body traits are correlated in this breed. Therefore, we hypothesized that there is a significant relationship between InDel variants within the *CFAP43* gene and body traits in SBWC goats. Herein, we first investigated the association between three InDel variant loci (L-13, L-16, and L-19 loci) within *CFAP43* and body traits in SBWC goats (n = 1827). Analyses revealed that the L-13, L-16, and L-19 loci were significantly associated with chest depth, four body traits, and three body traits, respectively. The results of this study are in good agreement with those previously reported and could provide useful molecular markers for the selection and breeding of goats for body traits.

## 1. Introduction

The cilia- and flagella-associated protein 43 (*CFAP43*) gene, also known as *HYDNP1*, *WDR96*, or *SPGF19*, is a protein-coding gene that is widely present and expressed in gonadal organs [1]. The *CFAP43* gene is involved in the formation of cilia. Primary cilia are hair-like immobile organelles with specific membrane receptors, including Hedgehog signaling receptors. Cilia organized in pre-osteoblasts enable bone formation by mediating Hedgehog signaling and promoting cell differentiation into osteoblasts [2]. Reducing the number and length of cilia inhibits the differentiation of primary osteoblast precursors. For example, diabetes leads to the loss of cilia in osteoblasts, resulting in defective diabetic fracture healing [3]; thus, cilia are essential for bone formation. Additionally, when cilia are present in the digestive system, they manipulate and collect food in liquids [4]. The melanin-concentrating hormone system is heavily localized to the primary cilia of neurons and is engaged in a number of areas, including energy balance and food intake [5]; thus, cilia are also important for food intake and energy metabolism.

Osteogenesis [6] and energy metabolism [7] can directly affect animal growth and development. Therefore, cilia play an essential role in the body traits of animals. *CFAP43* is expressed in tissues carrying motile cilia and acts as a target gene for *FOXJ1*, which is essential for inducing motile cilia formation [1]. *CFAP43* plays a role in spermatogenesis [8], and a 6-bp InDel mutation in the *CFAP43* gene can be used as a useful molecular marker for reproductive trait selection in goats [9]. In addition, a novel 4-bp insertional mutation in intron 7 of this gene is significantly associated with litter size in goats (*p* = 0.001) [10]. Therefore, we hypothesized that the *CFAP43* gene is closely associated with growth as it influences cilia formation, which affects energy metabolism and bone formation.

Body traits are quantitative traits controlled by micro-effective polygenes and are more difficult to select than qualitative traits; thus, they require more accurate and efficient selection methods [11]. Quantitative traits are determined and influenced by both environment and genetics. Therefore, it is feasible to select animals for breeding via genetic molecular marker-assisted breeding. Insertion/deletion mutation (InDel) in the DNA sequence is caused by the insertion or deletion of a small fragment; the technique of detecting InDel variants is a type of molecular marker technology, which has the advantages of simplicity, operability, and low testing cost. InDel variants have several applications in livestock selection. For example, an InDel within the *SPAG17* gene was identified in 1520 cattle from eight breeds, and the insertion/deletion (ID) genotype was found to be the ideal genotype for traits such as height and body slope length [12]. The InDel variant of the *SIRT4* gene affects body traits in beef cattle [13]. In sheep, InDels of *IGF2BP1* and *PLAG1* also influence growth traits [14,15]. In goats, two InDel variants within the *SNX29* gene had significant effects on the growth performance of 1759 goats [16]. In Shaanbei white cashmere (SBWC) goats, the InDel of the *HIAT1* gene has significant effects on chest depth, chest circumference, body length, and hip circumference [17]. Additionally, intron InDel variants within the *CDC25A* gene [18], the *FTO* gene [19], and the *CPT1a* gene [20] are associated with body traits. The *CFAP43* gene in goats has 38 exons and is located on chromosome 26 [9]. A 4-bp insertion in the *CFAP43* gene affects litter size in goats [10], and litter size is closely related to body traits in animals [21]. However, few studies have been reported on the association of InDel of the *CFAP43* gene with growth phenotypes.

Here, we investigated the associations of three InDel loci (L-13, L-16, and L-19) of the *CFAP43* gene with body traits in goats, which will provide useful molecular markers for the selection and breeding of goats for body traits.

## 2. Materials and Methods

### 2.1. Collecting Animal Samples, Recording Phenotypes, Isolating DNA

The SBWC goats were reared on the goat farm of the Shaanbei White Cashmere Goat Engineering and Technology Research Centre, Yulin College, Shaanxi Province, China. A total of 1827 mature female goats were randomly selected from each farm. The selected goats were approximately 2 years old and had been kept under the same diet and environmental conditions (all goats were healthy) [9,10]. Body traits such as chest depth, body length, chest width, cannon circumference, back height, body length, and chest circumference were recorded; measurements were taken by an assistant who pulled the goat onto a flat surface and stabilized it so that it stood in a natural position while taking the measurements, using soft rulers and measuring sticks [22].

Ear tissues from 1827 adult female goats were collected for genomic DNA extraction. Genomic DNA was isolated from the ear tissue using a standard protocol [23,24]. Genomic DNA quality was measured using a Nanodrop 2000 spectrometer (Thermo Scientific, Waltham, MA, USA) and diluted to a working concentration (20 ng/μL) for the detection of genetic variants. DNA was extracted using a high-salt extraction method [25,26]. In addition, pre-experiments were carried out, and the pooled DNA samples from each of the 30 goats were tested using polymerase chain reaction (PCR) to analyze the polymorphism of loci [23,24].

### 2.2. Primer Design, PCR Amplification, and InDel Detection

Three pairs of primers [9,10] were designed for amplification, based on the goat *CFAP43* gene sequence (NC_030833. 1) using Primer 5.0 software (Version 5.0, Premier Biosoft International, Palo Alto, CA, USA) (Table 1).

The PCR experiments were conducted in a 13-μL reaction system. The reaction system consisted of 0.5 µL of primers (forward and reverse), 6.5 µL of 2 × Eco Taq PCR Super Mix, 0.8 µL of DNA, and 4.7 µL of H_2_O. The PCR amplification process was described previously [27]. The PCR products were examined via electrophoresis on 3.5% agarose gel using ethylenediamine staining, and the PCR products were sequenced. Finally, the genotypes were identified. There were three genotypes, with a longer band representing genotype insertion/insertion (II). A shorter band indicated genotype deletion/deletion (DD). Two or three (homoduplex) bands indicated genotype insertion/deletion (ID) [9,10].

### 2.3. Statistical Analysis and Cluster Analysis

Association analysis of the *CFAP43* gene with body traits was performed using SPSS (version 25.0, IBM Corporation, New York, NY, USA). Analyses for two genotypes were performed using an independent sample *t*-test (L-13 locus), and those for three genotypes were performed using one-way ANOVA (L-16 and L-19 loci). In addition to the analysis of the correlation between the three loci and each body trait, the effects of the three loci on body traits at different litter sizes were also analyzed. The following basic linear model was used: Y_ijkmn_ = μ + G_i_ + S_j_ + M_k_ + I_m_ + e_ijkmn_, where Y_ijkmn_ is the phenotypic value of the body trait, μ is the average value of the population, G_i_ is the influence of genotype, S_j_ is the effect of age, M_k_ is the maternal effect, I_m_ is the gene interaction effect, and e_ijkmn_ is the random error. A value of *p* < 0.05 indicates statistical significance. Pie charts of genotype frequencies were drawn according to the frequencies of the genotypes (L-13, L-16, and L-19) [9,10].

A clustering tree of different species on the *CFAP43* gene was drawn using MEGA-X software (https://www.megasoftware.net/) accessed on 21 July 2022 [28]. The distribution of InDel loci on the *CFAP43* gene was mapped using Exon-Intron Graphic Make (http://www.wormweb.org/exonintro) accessed on 22 July 2022. The full sequence of the *CFAP43* gene was downloaded from the National Center for Biotechnology Information website (https://www.ncbi.nlm.nih.gov/) accessed on 22 July 2022, and the locations of the identified loci [9,10] were from the Ensembl database (http://asia.ensembl.org/index.html) accessed on 22 July 2022.

## 3. Results

### 3.1. InDel Variants of the Goat CFAP43 Gene

**Figure 1:**
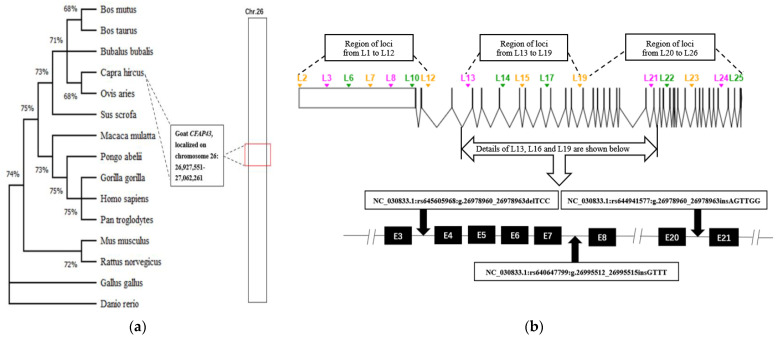
Species clustering of *CFAP43* gene and distribution of InDel variant loci. (**a**) Clustering trees of different species on the *CFAP43* gene; (**b**) distribution of identified InDel loci in the *CFAP43* gene of goats. Note: The percentages on the branches in (**a**) show the percentage of data coverage of the internal nodes. The colored triangles in (**b**) represent the identified InDel loci; black boxes represent exons of the goat *CFAP43* gene; and double slashes indicate omitted nucleotide sequences.

Figure 1a shows that *CFAP43* is widespread in a variety of animals, and the *CFAP43* gene sequence of goats is relatively similar to that of Ovis aries, Bos mutus, and Bos taurus. Among the 26 InDel loci within the *CFAP43* gene in SBWC goats, three loci were polymorphic (Table 2). Of these three loci, L-13 and L-16 were located in introns 3 and 7, respectively. The L-13 locus had two genotypes (II and ID) with a 4-bp deletion variant of TCCA. The L-16 locus had three genotypes (II, ID, and DD), with a 4-bp insertion variant of GTTT. Sequencing and electropherograms have been described previously [10]. The L-19 locus had three genotypes (II, ID, and DD), with a 6-bp deletion variant of AATTCT [9].

### 3.2. Gene Frequencies, Allele Frequencies, and Genetic Parameters

**Figure 2:**
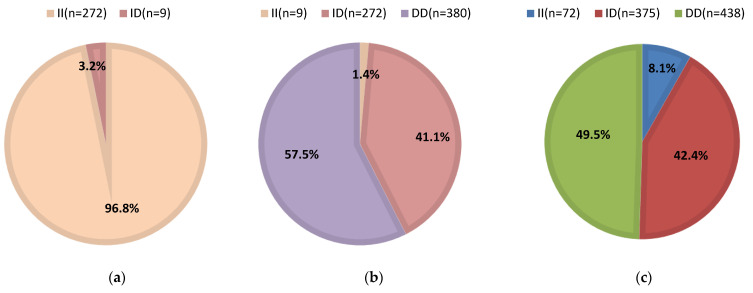
Pie charts of genotype frequencies for three InDel variant loci within the *CFAP43* gene. (**a**) Genotype frequencies at the L-13 locus; (**b**) genotype frequencies at the L-16 locus; (**c**) genotype frequencies at the L-19 locus.

Of the 281 individuals tested, the “I” gene frequency at locus L-13 was 98.4% and the “D” gene frequency was 1.6%. This locus was at a low genetic polymorphism level and was compatible with the Hardy–Weinberg equilibrium. Among the 661 individuals tested, the percentages of genotype II, ID, and DD at the L-16 locus were 1.4%, 44.1%, and 57.5%, respectively (Figure 2). This locus was in moderate genetic polymorphism and did not conform to the Hardy–Weinberg equilibrium. The results of linkage disequilibrium analysis at L-13 and L-16 showed that the two loci were not linked [10]. Among the 885 individuals tested, the frequencies of genotypes II, ID, and DD at L-19 were 8.1%, 42.4%, and 49.5%, respectively (Figure 2). This locus showed moderate genetic polymorphism, consistent with the Hardy–Weinberg equilibrium [9].

### 3.3. Association between Genotypes and Body Traits

At the L-13 locus, goats with genotype II had a significantly greater chest depth than those with genotype ID (*p* = 0.031) (Table 3).

At the L-16 locus, DD genotype goats were significantly longer than II genotype goats (*p* = 0.026); ID or DD goats had significantly larger back height and chest width than II goats (back height: *p* = 0.023; chest width: *p* = 0.026); DD goats had a significantly greater cannon circumference than ID or II goats (*p* = 0.016). At the L-19 locus, the ID or DD genotype goats had significantly greater body length, chest circumference, and cannon circumference than II genotype goats (body length: *p* = 0.010; chest circumference: *p* = 0.031; cannon circumference: *p* = 0.001) (Table 3).

At L-16, the ID or DD genotype goats had a significantly larger chest width than II genotype goats when the litter size was 1 (*p* = 0.038). When the litter size was 2, the ID or DD goats had a significantly greater cannon circumference than II goats (*p* = 0.021). At L-19, when the litter size was 3, II goats were significantly taller than ID goats (*p* = 0.004) (Table 4).

## 4. Discussion

Herein, the relationships between three InDel polymorphisms (L-13, L-16, and L-19) in the goat *CFAP43* gene and body traits were analyzed in 1827 local goats. The results showed that L-13 was related to chest depth. L-16 was associated with body length, chest width, cannon circumference, and back height. L-19 was associated with body length, chest circumference, and cannon circumference. The results were interpreted in terms of gene function, relationship between litter size and growth, and effect of intron variation as follows:

First, previous reports have indirectly indicated that the *CFAP43* gene is related to linear body measurements. The *CFAP43* gene is closely associated with cilia [29], and cilia affect body traits in both osteogenesis and energy metabolism. The absence of cilia can lead to shortened limbs [30] and disturbed lipid metabolism [31] and affect body homeostasis [32]. In addition, cilia on the cell surface are closely associated with the movement of certain cells. They can sense the external environment and may be involved in material transport and intracellular signaling, which then affects the growth and development of the animal body [33]. In conclusion, the *CFAP43* gene may influence body traits through energy metabolism and bone formation, as well as cell repair, signaling, and motility.

Second, InDel variants in *CFAP43* affect litter size. Correlations between litter size and body traits have been reported in goats [21], and genes have been found to affect both litter size and growth, in many cases: for example, *IGF2-BP2* (insulin-like growth factor 2 mRNA-binding protein 2) plays a key role in the development of diabetes and animal growth and is also a candidate gene for litter size in goats [34]. Mistranslation mutations in the *POU1F1* gene affect litter size and body traits in SBWC goats [35]. *PITX2* [36], *Runx2* [37], *PRNT* [38], and many other genes have been shown to affect both litter size and body traits. There have been two main studies on the effects of the *CFAP43* gene on litter size. One study [10] reported the effect of three InDel variants (*CFAP43*-P1, *CFAP43*-P2, and *CFAP43*-P3) on litter size in SBWC goats; the litter size of individuals with the DD genotype was significantly larger than that of individuals with the ID genotype at *CFAP43*-P3 locus. The other study [9] reported that a 6-bp deletion (L-19 locus) of the *CFAP43* gene significantly affected litter size; goats with the DD or ID genotype had significantly larger litter sizes than those with the II genotype. In our trial, the body length, chest width, cannon circumference, and back height at the L-16 locus were greater in individuals with the DD genotype. Body length, chest circumference, and cannon circumference were also greater in individuals with DD genotype at the L-19 locus. The results were consistent with those previously reported for litter size [9,10]; DD is the ideal genotype for both litter size and the above-mentioned body traits. Therefore, the results of this study provide a valid molecular marker locus for simultaneously improving growth and litter size. *CFAP43* is highly expressed in the oviduct, and mutations in *CFAP43* affect litter size by changing the cilia structure and thus egg function and fertilized egg transport [39,40].

Finally, InDel variants can directly affect gene structure and function [41]. When InDel variants occur in exons, they can directly affect mRNA translation. When they occur in introns, they may affect gene expression by influencing the binding of transcription factors to genes [42,43]. InDel variants in introns should not be ignored; many InDels located in intron regions of genes significantly affect animal production traits. For example, variants in the introns of *SNX29* [16], *IGF2BP* [14], and *AKAP12* [44] significantly affect body traits in animals. Similar to the results of the previous studies, the three InDel loci in this trial were also located in introns, and they significantly correlated with body traits.

This study is the first to investigate the relationship between the InDel variants of the *CFAP43* gene and body traits in goats, complement the function of the gene, and provide a theoretical basis for the application of this gene in the improvement of body traits in goats.

## 5. Conclusions

InDel variants at L-13, L-16, and L-19 of the *CFAP43* gene were significantly associated with body traits in the SBWC goat population, suggesting that these loci of the *CFAP43* gene can be used as genetic markers to simultaneously improve growth and litter size in goats.

## Figures and Tables

**Table 1:** PCR primers used to amplify target sequences.

Primers	Sequences (5′-3′)	Loci	Length (bp)
L-13	F: GGGTCCTTGTTGGTGGTAAACA R: GGATTGTTTTTACTTTCTGCCTCG	intron variant	157/153
L-16	F: TGCAATGGACTACCTCTTTCTACA R: TGCTTTCTCCAAGATCATCATCAC	intron variant	169/173
L-19	F: GGACAGAGAGACAGAGTTTCAGGT R: CAGACTCCCCTATCTTCAGATTA	intron variant	168/162

**Table 2:** Summary of InDel site status within the *CFAP43* gene in Shaanbei white cashmere goats.

Rs Number	Location	NP	NT	Polymorphism	Citations
rs637928228	g.26924232_26924254del	P1	L1	No polymorphism	[9]
rs645605968	g.26927779_26927789del	P8	L2	No polymorphism	[9]
rs640647799	g.26935998_26936011del	P2	L3	No polymorphism	[9]
rs660531175	g.26936406_26936419del	P3	L4	No polymorphism	[9]
rs670883772	g.26936813_26936814ins	P9	L5	No polymorphism	[9]
rs649997325	g.26942655_26942656ins	P10	L6	No polymorphism	[9]
rs637959012	g.26949228_26949229ins	P11	L7	No polymorphism	[9]
rs669535680	g.26955481_26955486del	P12	L8	No polymorphism	[9]
rs638243880	g.26956800_26956804del	P13	L9	No polymorphism	[9]
rs660347164	g.26961949_26961955del	P14	L10	No polymorphism	[9]
rs646148976	g.26962608_26962611del	*CFAP43*-P1	L11	No polymorphism	[10]
rs639683881	g.26966821_26966830del	P15	L12	No polymorphism	[9]
rs663035021	g.26978960_26978963del	*CFAP43*-P2	L-13	Polymorphism	[10]
rs646466463	g.26989558_26989563del	P16	L14	No polymorphism	[9]
rs667434652	g.26995455_26995460dup	P17	L15	No polymorphism	[9]
rs642797226	g.26995509_26995512dup	*CFAP43*-P3	L-16	Polymorphism	[10]
rs652206372	g.27003007_27003016del	P18	L17	No polymorphism	[9]
rs638859240	g.27005906_27005910del	P19	L18	No polymorphism	[9]
rs640685693	g.27012990_27012996dup	P20	L-19	Polymorphism	[9]
rs669411788	g.27016234_27016239del	P21	L20	No polymorphism	[9]
rs642762371	g.27034710_27034711ins	P22	L21	No polymorphism	[9]
rs640237668	g.27039504_27039505ins	P4	L22	No polymorphism	[9]
rs644941577	g.27047056_27047079del	P5	L23	No polymorphism	[9]
rs655570881	g.27056053_27056058dup	P23	L24	No polymorphism	[9]
rs667953078	g.27063791_27063805dup	P6	L25	No polymorphism	[9]
rs666915769	g.27064156_27064174del	P7	L26	No polymorphism	[9]

Note: NP: Naming in published articles, NT: Naming in this article.

**Table 3:** Associations between InDel variants in *CFAP43* gene and body traits of the Shaanbei white cashmere goats (Mean ± Standard error). Different letters indicate significant differences at *p* < 0.05.

Loci	Body Traits (cm)	II	ID	DD	*p*-Values
L-13	Chest Depth	27.62 ± 0.15 (n = 269)	25.91 ± 0.93 (n = 11)	-	0.031
L-16	Body Length	64.39 ± 2.74 ^a^ (n = 9)	64.84 ± 0.36 ^ab^ (n = 268)	65.89 ± 0.30 ^b^ (n = 376)	0.026
	Chest Width	15.94 ± 0.85 ^a^ (n = 9)	17.65 ± 0.15 ^b^ (n = 269)	17.78 ± 0.12 ^b^ (n = 378)	0.026
	Cannon Circumference	7.69 ± 0.13 ^a^ (n = 9)	7.92 ± 0.06 ^a^ (n = 270)	8.11 ± 0.04 ^b^ (n = 375)	0.016
	Back Height	53.87 ± 1.78 ^a^ (n = 4)	58.66 ± 0.34 ^b^ (n = 157)	58.29 ± 0.3 ^b^ (n = 157)	0.023
L-19	Body Length	64.28 ± 0.85 ^a^ (n = 69)	66.33 ± 0.32 ^b^ (n = 351)	66.70 ± 0.29 ^b^ (n = 417)	0.010
	Chest Circumference	86.85 ± 1.29 ^a^ (n = 69)	89.50 ± 0.47 ^b^ (n = 351)	89.92 ± 0.43 ^b^ (n = 417)	0.031
	Cannon Circumference	7.81 ± 0.12 ^a^ (n = 69)	8.22 ± 0.47 ^b^ (n = 352)	8.28 ± 0.04 ^b^ (n = 416)	0.001

**Table 4:** Associations of the loci of the *CFAP43* gene with body traits in Shaanbei white cashmere goats at different litter sizes (mean ± standard error (se)). Different letters indicate significant differences at *p* < 0.05.

Loci; Litter	Body Traits (cm)	Observed Genotypes (Mean ± SE)			*p*-Values
		II	ID	DD	
L-16; single lamb	Chest Width	14.30 ± 0.17 ^a^ (n = 5)	17.05 ± 0.2 ^b^ (n = 155)	17.33 ± 0.2 ^b^ (n = 176)	0.038
L-16; twin lamb	Cannon Circumference	7.55 ± 0.16 ^a^ (n = 4)	8.46 ± 0.07 ^b^ (n = 109)	8.50 ± 0.05 ^b^ (n = 198)	0.021
L-19; triple lamb	Body Height	65.00 ± 1.18 (n = 7)	57.75 ± 1.31 (n = 4)	-	0.004

## Data Availability

Data are available upon request from corresponding author.
